# Quantitative SERS-Based Sandwich-Hybridization Assay for Nucleic Acid Detection

**DOI:** 10.1021/acsomega.5c01977

**Published:** 2025-10-28

**Authors:** Kosar Shahsavar, Amr Mostafa, Dina Mahdi-Joest, Anton S. Zverev, Sergio Kogikoski, Morteza Hosseini, Ilko Bald

**Affiliations:** † Institute of Chemistry, Hybrid Nanostructures, 26583 University of Potsdam, Karl-Liebknecht-Str. 24-25, Potsdam 14476, Germany; ‡ Nanobiosensors Lab, Department of Nanobiotechnology and Biomimetics, School of Life Science Engineering, College of Interdisciplinary Science and Technology, 48425 University of Tehran Tehran 1439817435, Iran

## Abstract

Surface-enhanced Raman spectroscopy (SERS) has great potential for quantifying biological molecules, particularly DNA. In this study, we developed a SERS-based sandwich-hybridization assay for detecting DNA corresponding to viral (SARS-CoV-2) RNA using commercially available components and standard bioconjugation procedures; hence, no sophisticated synthesis steps were required. The assay employs TAMRA-labeled DNA conjugated to gold nanoparticles as nanoprobes and biotin-labeled surface DNA strands. Upon hybridization with the target analyte, the complete sandwich structure forms and is captured on a neutravidin-coated glass via the avidin–biotin interaction. The observed Raman signals originate from the TAMRA reporter dye, leveraging signal enhancement from both resonance and plasmonic effects, enabling the sensitive detection of the target analyte. Quantification of target DNA was performed using both a conventional single-point peak-height spectral analysis and a binary image-based analysis, with the latter providing a reliable evaluation of the assay’s performance. Additionally, atomic force microscopy (AFM) was employed to characterize the samples, and the strong correlation between AFM and SERS results confirms the accuracy and reliability of this approach.

## Graphical abstract

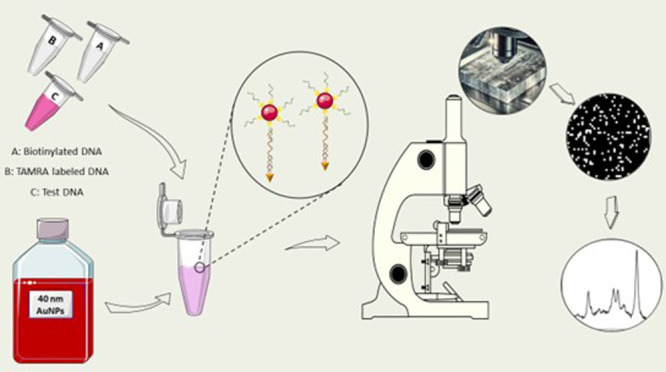

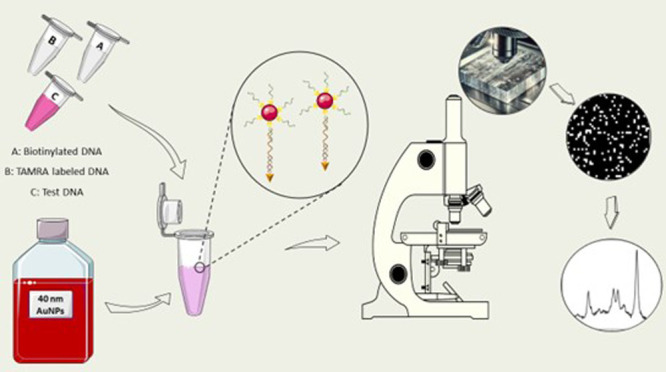

## Introduction

Public health and human life are increasingly threatened by the emergence and reemergence of fatal viral diseases, such as COVID-19, caused by the severe acute respiratory syndrome coronavirus type 2 (SARS-CoV-2).^1^ Nucleic acid amplification tests (NAATs), such as real-time reverse-transcription polymerase chain reaction (rRT-PCR), are the gold standard for detecting viral infections due to their high sensitivity and specificity.^2^ However, RT-PCR has limitations, including complex primer design and the risk of false-negative results.^3^ These challenges highlight the need to develop new and more efficient methods for viral DNA detection.

Surface-enhanced Raman scattering (SERS) is an inelastic light scattering of a target analyte, enhanced by the close proximity of a roughened metal surface or metal nanoparticles.^4^ SERS has been applied in ultrahigh-sensitive analysis,^5,6^ molecular fingerprinting,^7^ single-molecule detection,^8,9^ and multiplex sensing methods.^10,11^ However, quantitative SERS measurements remain challenging due to poor reproducibility and signal fluctuations.^12,13^ The SERS signal is mainly influenced by the molecule of interest, the SERS substrate, their interaction, and the instrument used. Establishing constant values for these instrumental variables could enhance the reliability of SERS data for quantitative measurements.^14,15^ Two additional challenges are ensuring homogeneous SERS substrates and consistent molecule–substrate interactions.^16,17^ SERS nanoprobes, which combine recognition and signal transduction functions, effectively address these challenges and provide a promising approach for detecting biomolecules such as DNA.^18^ In DNA analysis, the inherently low Raman cross-section makes direct detection difficult.^19^ Using a sandwich-hybridization assay with SERS nanoprobes compensates for weak Raman signals and reduces signal variation, enhancing sensitivity and specificity in DNA detection.^20^

In 1999, the first SERS-based sandwich assay was reported.^21^ Since that time, SERS-based sandwich assays have been widely employed owing to their distinct advantages: narrow Raman peaks enabling multiplex analyte detection, high sensitivity resulting from large enhancement factors, and reduced susceptibility to photobleaching compared with fluorescence. Building on this foundation, various SERS-based sandwich hybridization assays have been developed for the detection of specific DNA sequences.^22,23^ Khalil et al. designed a dual nanoplatform for qualitative and quantitative detection of DNA, incorporating a graphene oxide-gold nanorod (GO-AuNR) functionalized with a capture probe, along with signal-probe-conjugated gold nanoparticles (AuNPs). The fabricated biosensor demonstrated a limit of detection (LOD) as low as 100 aM.^24^ Furthermore, a magnetically responsive SERS platform with a tunable hot spot was created, consisting of an AuNP/rGO hybrid substrate with DNA probe 1, superparamagnetic iron oxide nanoparticles (SPIONs) with DNA probes 2 and 3, and a Raman reporter linked to DNA 4.^25^ This platform shows a linear Raman response to DNA concentrations from 1 fM to 1 nM, with a detection limit of 1 fM. Therefore, current research focuses on the development of plasmon-active substrates using indirect SERS strategies.^26^

While the primary challenges in SERS have been discussed, an equally critical consideration is the challenge of accurately measuring and analyzing SERS signals. To address these challenges, we implemented two analytical approaches: the single-point peak-height method and the digital mapping method. The single-point approach evaluates the intensity at a specific Raman peak, but its accuracy can be compromised by background fluctuations, noise, and peak shifts, particularly at low concentrations.^27^ In contrast, digital mapping converts SERS signals into binary data, offering improved sensitivity at low concentrations by reducing the impact of intensity fluctuations.^28^ Comparing both methods in our SERS-based DNA assay allowed us to identify the most accurate and practical solution for quantitative DNA detection.

This study investigates a sandwich-hybridization SERS assay system for rapid pandemic disease detection, utilizing commercially available components and well-established laboratory methods to evaluate its effectiveness and identify potential challenges. We developed an SERS-based sandwich-hybridization assay using TAMRA-labeled DNA-functionalized gold nanoparticles as SERS nanoprobes for CoV-DNA detection. The SERS nanoprobe comprises a gold nanoparticle (AuNP), TAMRA dye as a Raman reporter, and a complementary DNA strand (Figure 1). The test DNA is recognized by two capture strands: a partially complementary DNA strand on the nanoprobe and a biotin-labeled complementary surface DNA strand. In the presence of an analyte, the whole sandwich structure is formed and deposited on neutravidin-coated glass via an avidin–biotin interaction. The collected signals from the nanoprobes are used to quantify the test DNA concentration based on conventional and digital methods. The linearity of the logarithmic plot is improved using the digital method compared with the conventional method.

**Figure 1:**
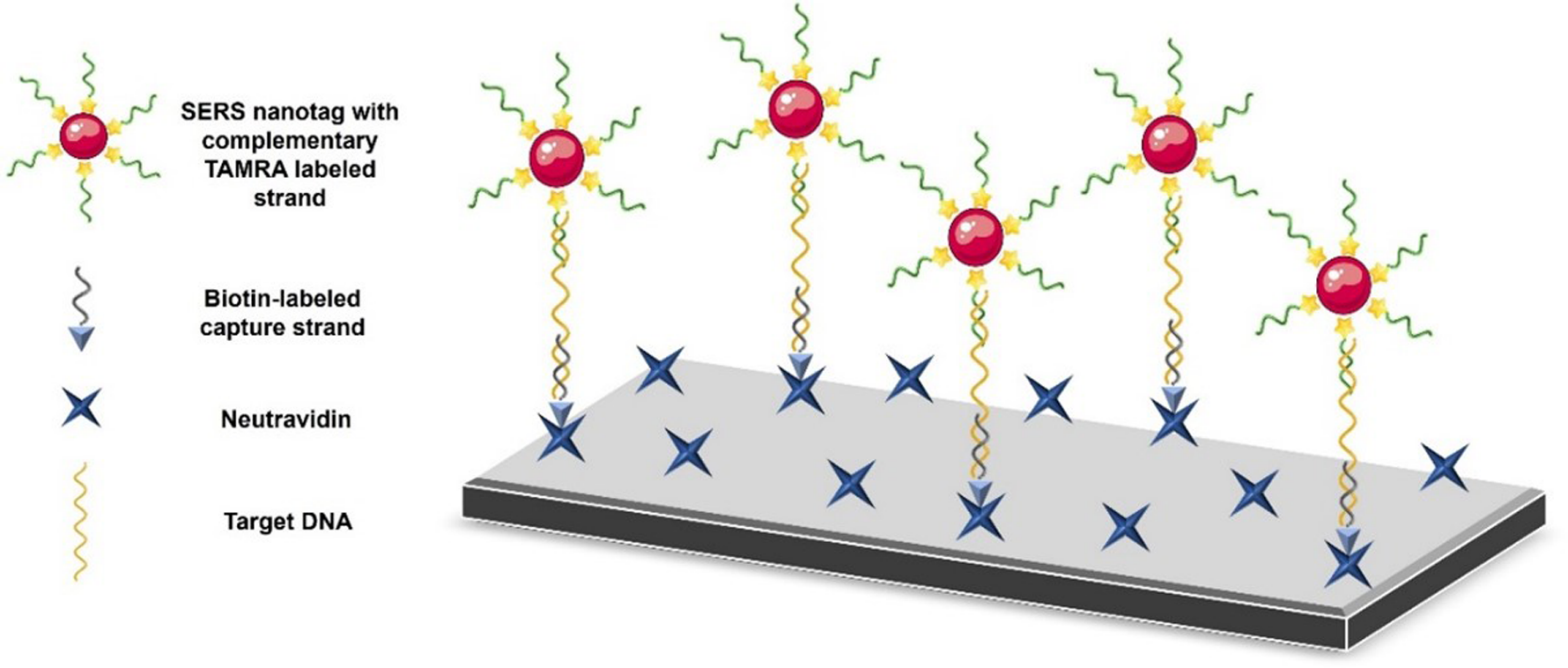
Schematic representation of CoV-DNA detection by SERS. A target DNA strand (in yellow) is captured by a dye-labeled complementary probe (in green) and a biotin-labeled capture probe (in gray , resulting in the formation of a sandwich structure. The complex is immobilized on a glass surface via biotin–avidin binding, and the SERS signal is predominantly derived from the TAMRA reporter for quantitative analysis.

## Methods and Materials

Gold (Au nanosphere, citrate-coated, 40 nm) nanoparticles were purchased from nanoComposix (San Diego, CA, USA). Tris­(2-carboxyethyl)­phosphine (TCEP) was acquired from Sigma-Aldrich Chemie GmbH (Munich, Germany). DNA strands were acquired from Metabion International AG (Planegg, Germany), and the sequences are listed in Table 1. TAMRA (carboxytetramethylrhodamine) is a widely used fluorescent dye for oligonucleotide labeling. In this study, TAMRA-labeled DNA strands were employed as Raman reporters in the SERS nanoprobes due to their strong and stable Raman signals and their well-established compatibility with DNA conjugation. The test DNA sequence, called CoV-DNA, was selected from the viral S-gene (surface glycoprotein) of SARS-CoV-2. The complementary sequence in the test strand is highlighted in italics and underlined, and the errors are shown in blue in the mismatched strand. Covalently coated NeutrAvidin glass slides were purchased from PolyAn (PolyAn GmbH, Berlin, Germany).

**Table 1:** List of DNA Sequences Used in the Study Includes Relevant Details on Modification and Complementarity

**Name**	**Sequence**	**Length**	**5′ Mod**	**3′ Mod**
**Nanoparticle strand**	**TCAAGGTCACTACCACTAGT**	**20**	**Thiol-C6-Tamra**	
**Surface strand**	**AATAATAAGAAAATAAACAT**	**20**		**Biotin**
**Test strand**	***ATGTTTATTTTCTTATTATT*** **TCTTACTCTC** ***ACTAGTGGTAGTGACCTTGA***	**50**		
**Mismatch sequence strand**	**ATGTTTATTGGGTTATTATTTCTTACTCTCACTAGTGGTCCCGACCTTGA**	**50**		

### Synthesis of SERS Nanoprobes

Nanoparticle coating methods vary, such as salt aging,^29,30^ pH alteration,^31^ and the freezing- directed method.^32^ In this study, AuNPs were coated with TAMRA and thiol-double-labeled DNA using a freezing technique. This approach has been reported to be straightforward and applicable for all DNA sequences, resulting in a dense DNA layer after sample thawing.^33,34^ First, 400 μL of 40 nm AuNP stock solution (0.14 nM) was centrifuged (once at 5000 rpm, 5 min), and the supernatant was removed. Then, the pellet was redispersed in Milli-Q water to a final volume of 25 μL. Separately, 1 μL of 100 mM TCEP solution was incubated with 4 μL of thiol-modified DNA (100 μM) for 10 min at room temperature (RT) to cleave the disulfide bond and activate the thiol group in the DNA strand. Later, 5 μL of the cleaved DNA strand solution was added to the initially concentrated AuNP solution and frozen at −20 °C for at least 2 h. Finally, after sample thawing, the excess amount of coating strands was discarded by centrifugation (5000 rpm, 5 min at RT). The supernatant was removed, and the sample was resolubilized in 25 μL of water.

The gold nanoparticles were characterized using a scanning electron microscope (Hitachi S-4800) both before and after functionalization. For SEM analysis, the AuNP suspension was diluted 1:10 with ultrapure water. Aluminum rolling rings were used as sample holders, and black conductive carbon adhesive pads were affixed to the rings. A 3 μL aliquot of the diluted suspension was drop-cast onto the pads and allowed to dry completely at room temperature. Images were captured at 5 kV with a working distance of 5 mm.

### Preparation and Development of Sandwich Assay

In this study, the target DNA sequence is hybridized and sandwiched between a complementary strand on the nanoprobe and a biotin-labeled surface strand, which is then immobilized on a glass surface using the avidin–biotin interaction in order to conduct SERS measurements. Briefly, 25 μL of SERS nanoprobes were incubated with CoV-DNA (4 μL, different concentrations) and a biotin-labeled surface strand (4 μL, 100 μM) for 10 min at 37 °C. The biotin-labeled strand is used to capture CoV-DNA. Then, the SERS nanoprobe decorated with TAMRA-labeled DNA that is complementary to the rest of the CoV-DNA, can hybridize with it. The solution was then kept at RT for an additional 20 min. Following that, 5 μL of samples with 10 μL of buffer (1X TAE, 300 mM NaCl) were deposited on the avidin-coated glass surface for 7 min, rinsed with a water/ethanol solution, and then blow-dried.

### Characterization with AFM

The Atomic Force Microscopy (AFM) imaging was conducted using a FlexAFM instrument equipped with a C3000 controller from Nanosurf. Tapping mode was employed for imaging, conducted in air with a Tap 150 Al-G cantilever (Budget Sensors), operating at a resonance frequency of 150 kHz, with a nominal spring constant of 5 N/m and a 10 nm tip size. The glass slides prepared earlier were imaged over a 10 μm × 10 μm area at approximately 0.8 s/line scan speed, with 512 data points per line. The proportional gain (P-Gain) and integral gain (I-Gain) settings ranged from 1000 to 1500. The acquired images were processed using Gwyddion, an open-source software, involving specific steps such as leveling the data by mean plane subtraction and removing the polynomial background. AuNPs were manually counted, with height profiles used to confirm particle dimensions and distinguish them from surface artifacts. The particle numbers for each concentration were obtained by summing the counts from three images.

### SERS Measurements

Raman spectra were recorded using a LabRam HR Evolution Raman Microscope (HORIBA France SAS) equipped with a 532 nm laser. The laser power was maintained at 10% (100 mW at the laser head) throughout all the experiments. Exploiting SERS as a quantitative measurement technique presents challenges. One of the issues that may arise is sampling error, which can be mitigated by expanding the sample area. To address this, the mapped regions measured 200 × 200 μm^2^ with a 1 s acquisition time per pixel and a step size of 5 μm, resulting in a total of 1681 pixels (SERS spectra) per mapping area. The average spectrum obtained from 1681 individual spectra was used for further analysis.

Moreover, by using the objective with a smaller numerical aperture and increasing the laser illumination area, we can minimize the potential for sampling error. The laser spot size, defined as the diameter of the laser spot, is calculated by

laser spot diameter=1.22×λ/NA

where λ is the laser wavelength and NA is the numerical aperture of the objective. At a wavelength of 532 nm, the estimated laser spot diameters were 2.6, 1.3, and 0.7 μm for the 10×, 50×, and 100× objectives, respectively. Despite the advantages of a larger laser spot, there are certain drawbacks, such as lower excitation intensity and reduced collection efficiency in the optics. Therefore, the 50× objective (NA = 0.5) was chosen as a compromise for all experiments. For reference, the NA values of the other objectives used in this study were 100× = 0.90 and 10× = 0.25.

As part of data preprocessing and analysis, spikes and cosmic rays were removed from the raw spectra. Subsequently, a polynomial fitting approach was employed for baseline correction. Furthermore, spectra exhibiting abnormally high overall intensity due to nanoparticle aggregation were excluded by using a threshold-based approach. The processed data underwent sensitivity analysis through a conventional method that relied on the characteristic TAMRA peak (1652 cm^–1^, Figure 2F). Furthermore, the digital method was used to establish a quantitative relationship based on the pixel intensity from the Raman map.

**Figure 2:**
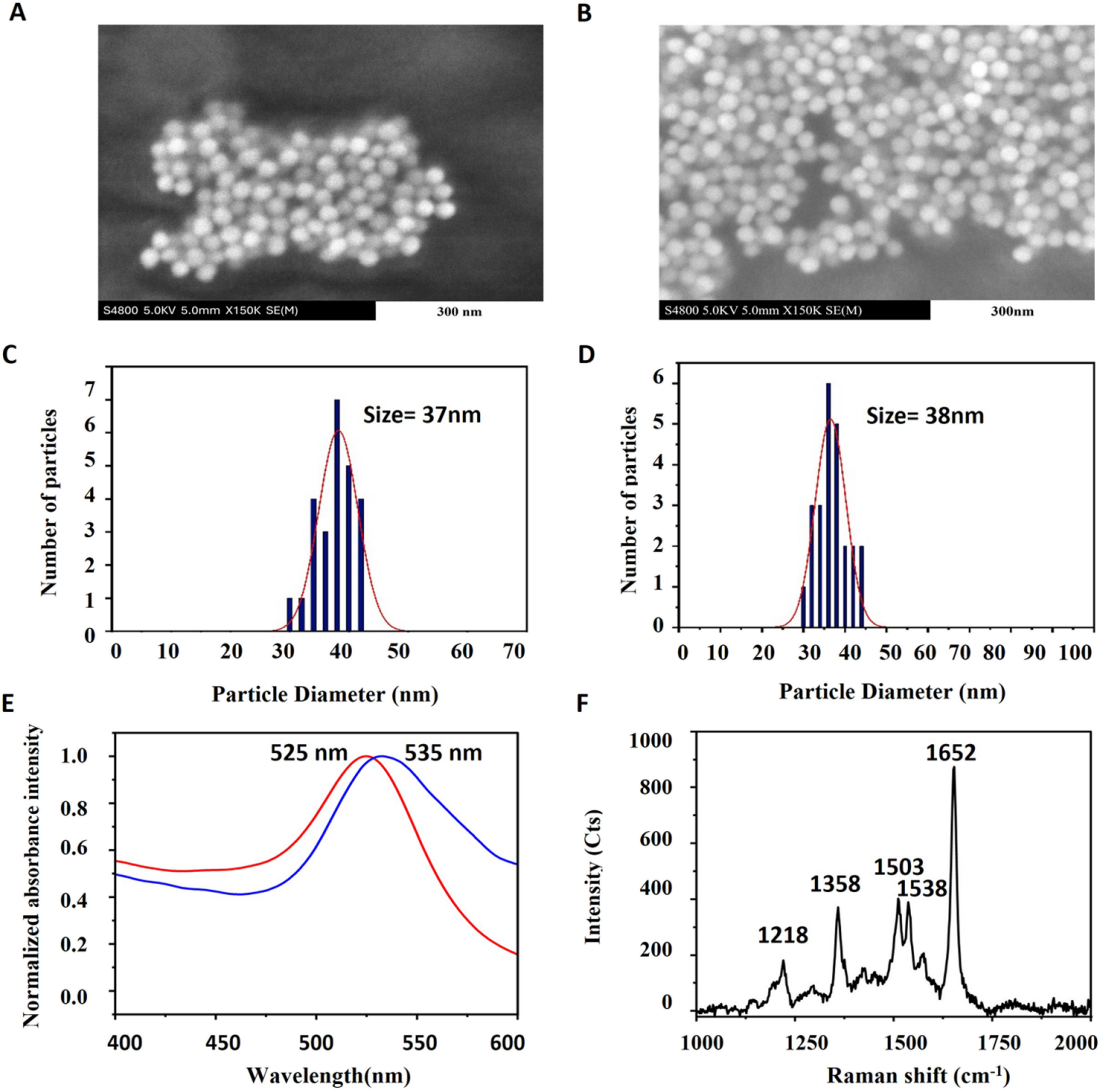
Scanning electron microscopy (SEM) image and size distribution of bare AuNPs (A, C) and coated Au nanoparticles (B, D). (E) UV–vis spectra of Bare AuNPs­(red) and coated AuNPs­(blue) (F) Raman spectra of Au nanoparticles­(aggregation) coated with TAMRA-oligo using a 532 nm laser.

## Results and Discussion

Figure 1 illustrates the SERS-based sandwich hybridization assay for CoV-DNA detection. Following hybridization, the biotinylated surface strand brings the sandwich complex onto neutravidin-coated glass slides. TAMRA serves as the primary Raman reporter in this approach, and its strong, distinct Raman cross-sections enable reliable quantification without the need for multivariate analysis. The main objective is to correlate the TAMRA signal with the CoV-DNA concentration using single-point peak-height spectral analysis and binary image-based analysis. This framework provides a robust basis for evaluating the assay sensitivity and its potential for quantitative nucleic acid detection.

### SERS Nanoprobe Characterization

The scanning electron microscopy (SEM) image in Figure 2 shows the morphology of gold nanoparticles before (a) and after (b) DNA functionalization. A uniform size distribution is found in the statistical histograms for bare nanoparticles (c) and functionalized nanoparticles (d). The average diameter of the particles is about 40 nm, as determined through Gaussian fitting. Figure 2E displays UV–vis characterization of nanoparticles in the visible range. A noticeable red shift in the LSPR peak is observed upon DNA modification: from 525 nm for bare Au nanoparticles to 535 nm for DNA-functionalized Au nanoparticles. The Raman spectrum of Au nanoparticles functionalized with a TAMRA-labeled probe is shown in Figure 2F, with the most intense peak selected as the characteristic peak for subsequent analysis. TAMRA is a photostable Raman reporter under resonant excitation (532 nm), with photobleaching occurring over several hundred seconds, well beyond the integration times used here.^35^

### Experimental Parameter Optimization

The assay procedure was systematically investigated and optimized by evaluating various experimental conditions to enhance the overall analytical performance of the biosensor. Atomic force microscopy (AFM) was employed to characterize the AuNPs, with the goal of maximizing the yield of isolated single AuNPs while minimizing aggregation. The first key focus was on the hybridization of the analyte with the biotinylated surface DNA and TAMRA-labeled functionalized AuNPs, a critical step in refining the sandwich assay. This step ensures specific and efficient binding of the target analyte to the nanoparticles, which is essential for accurate detection. To maximize the hybridization efficiency, various parameters were tested with the goal of optimizing the binding between the analyte and the two capture strands. Key factors, such as hybridization time and salt concentration (Table S1), were adjusted during the optimization process. Results (Figure S1A) indicated that the best hybridization condition involved a 10 min incubation at 37 °C, without 1× TAE 750 mM NaCl, followed by a 20 min incubation at room temperature. This condition resulted in the optimal ratio of single nanoparticles to aggregates, suggesting it as the most effective for the assay.

The second major objective was to systematically investigate the surface deposition process by varying the ionic concentration and incubation time. The aim was to maximize the yield of single AuNPs while minimizing nanoparticle aggregation and nonspecific binding. Aggregation can lead to uncontrolled SERS signal enhancement, resulting in increased variability and reduced reliability of quantitative measurements. The results (Figure S1B,C) showed that a 300 mM NaCl concentration and a 7 min incubation resulted in the highest yield of individual AuNPs with minimal aggregation. Additionally, nonspecific binding can arise from insufficient washing steps. To overcome this challenge, washing parameters were optimized. The results (Figure S1D) indicated that a one-time wash with a water/ethanol mixture effectively minimized nonspecific binding, improving biosensor performance and ensuring reliable, reproducible results.

### AFM Measurements

Atomic force microscopy (AFM) was used to confirm the successful functionalization of the surface of the assay with AuNPs. Initial images verified the presence of AuNPs (Figure 3A), indicating the correct operation of the immobilization system.

**Figure 3:**
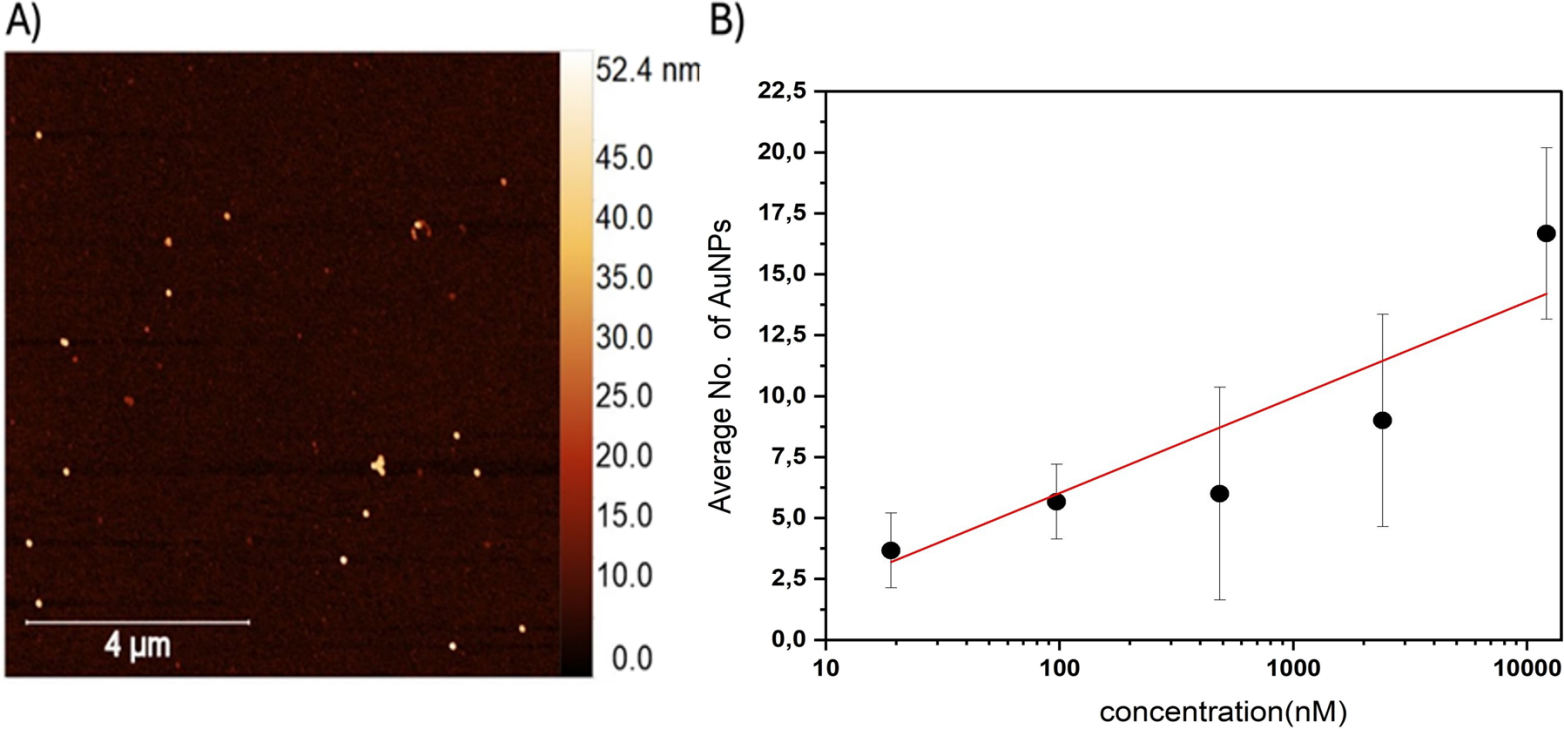
A) AFM image confirms the presence of AuNPs on the assay surface. B) Concentration-dependence plot of AuNP counts on the surface as a function of target DNA concentration (log scale). Error bars represent the standard deviation.

To establish a quantitative analysis method, AFM and Raman mappings were considered. An AFM-based concentration-dependence assay was performed over a target DNA concentration range of 0.019 μM to 12.1 μM (reported concentrations correspond to solution values used for sample preparation, before deposition and drying), demonstrating a positive correlation between target DNA concentration and the number of surface-bound AuNPs (Figure 3B). However, increasing target DNA concentrations also increased the standard deviation of observed AuNP counts, likely due to the inherently limited field of view in AFM imaging. It is worth noting that most NPs appeared as single particles, and only rarely were clusters of particles observed.

To improve data consistency and analysis efficiency, subsequent experiments utilized Raman spectroscopy. This technique allows for larger surface area scans, providing robust AuNP quantification. Additionally, Raman spectroscopy can measure the intensity of the TAMRA signal, potentially offering insights into binding efficiency and AuNP distribution. Furthermore, due to its noninvasive nature and minimal sample preparation requirements, Raman spectroscopy has been widely recognized as a promising tool for biomedical diagnostics and portable sensing platforms, suggesting its potential for future integration into point-of-care (POC) applications.^36^

### Assay Consideration Using Characteristic Peak Intensity

To verify the feasibility of quantitative detection using a SERS-based sandwich assay, Raman mapping was performed over a concentration range of 0.019 to 12.1 μM. Collected Raman maps were evaluated using two different methods: one based on characteristic peaks from spectral analysis and the other based on active areas from digital map analysis. For the spectral analysis, the intensity of the diagnostic peak was used to plot the calibration curve. The digital analysis considered the number of active pixels rather than the SERS signal intensity for the construction of the calibration curve. The observed SERS signals arose from the combined contributions of molecular resonance and plasmonic enhancement, with the interplay of these mechanisms generating the high signal intensities observed in the sandwich assay. To ensure consistent measurements, experiments were performed using the same batches of AuNPs with identical surface coverage, while critical factors affecting signal intensity during deposition were carefully optimized. Figure 4 shows the different Raman maps at the different concentrations of CoV-DNA. Average Raman spectra were taken from the entire Raman map area of each concentration, and then, the calibration curve was plotted based on the TAMRA peak (at 1652 cm^–1^) in each average spectrum. The TAMRA characteristic peak intensity was used for plotting the quantitation curve. Dots in the logarithmic plot correspond to the means of three measurements, and error bars show the standard deviation for each series. As can be seen from the figure, SERS signal intensity increased with an increase in CoV-DNA concentration from 0.019 to 12.1 μM. The limit of detection (LOD) can be defined as the lowest concentration that can be statistically distinguished. The LOD was calculated as 3.3 δ/*s*, where δ is the standard deviation of the blank sample and *s* stands for the slope of the calibration curve. Based on the above formula and values from the linear equation, the LOD was found to be 1.4 nM.

**Figure 4:**
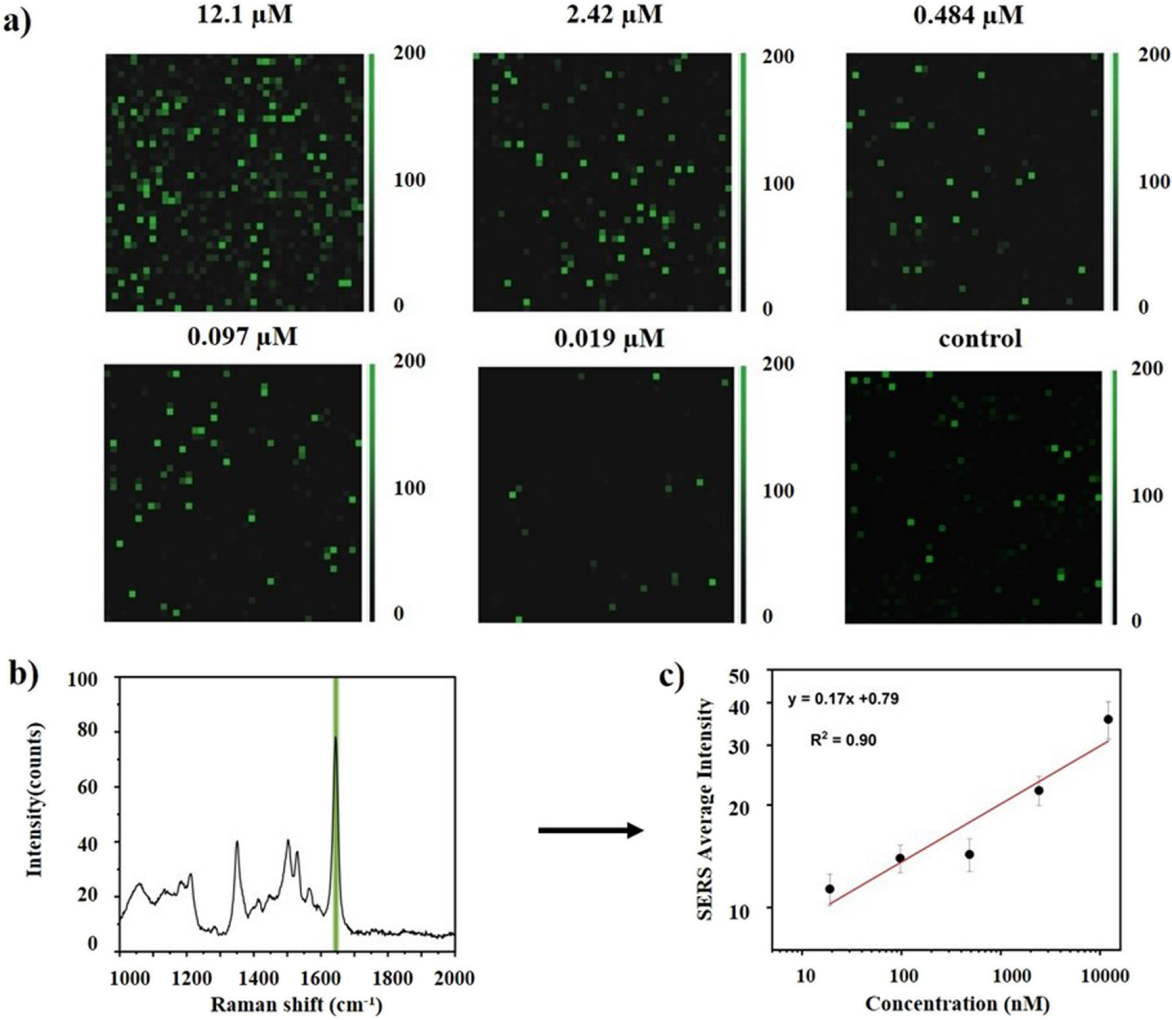
Detection and quantification of CoV-DNA using SERS nanoprobe intensity in a conventional method. (a) Raman color maps show intensity variations for different DNA concentrations. (b) Average spectra are extracted from each Raman map. (c) Logarithmic plot of the SERS intensity of the characteristic peak at 1652 cm^–1^ against the CoV-DNA concentration.

### AFM–Raman Correlation

We correlated the AFM results for each concentration to the SERS outcomes for the same concentration using the conventional method. As shown in Figure 5, the average number of particles in five AFM images for each concentration was plotted against the average intensity of the Raman spectrum from the same concentration. It is noteworthy that the mean intensity values were obtained after performing baseline correction. Background subtraction reduces signal variability and distribution heterogeneity. The coefficient of determination (R^2^) for the plotted linear regression was 0.99, indicating a high degree of correlation and an excellent fit of the model to the data. This result shows that the SERS response at different concentrations is linearly correlated to the number of SERS nanoprobes, indicating that the contribution of nanoparticle aggregates to the SERS signal intensity is minimal, i.e., the SERS signal is due to the presence of single nanoparticles.

**Figure 5:**
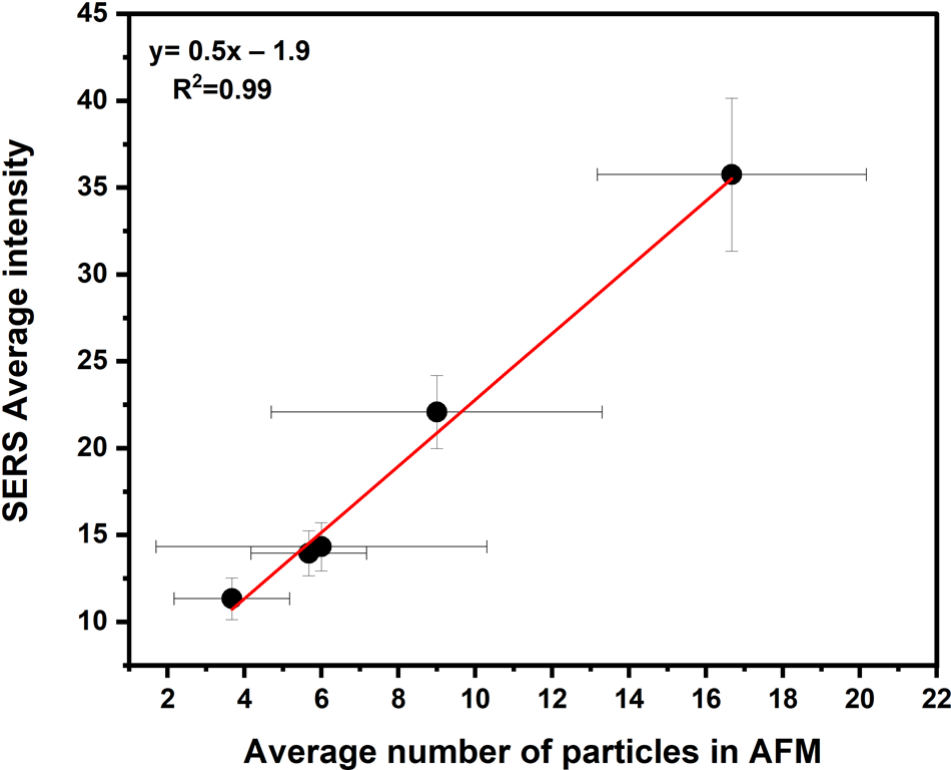
A linear relationship between the average number of AuNPs (SERS nanoprobes) present on a 100 μm^2^ surface, as determined by AFM, and the average SERS intensity obtained from a conventional Raman map with a size of 200 × 200 μm^2^ and a resolution of 5 μm. Error bars represent the standard deviation of repeated SERS measurements (*Y*) and AFM measurements (*X*) at each concentration.

### Evaluation of the Selectivity of the SERS-Based Sandwich Assay

The selectivity of the assay was evaluated through control experiments by analyzing the detection of mismatched sequences of the same length (50 bp) with variations in concentration. SERS responses were measured for CoV-DNA and mismatched DNA (III) under optimized conditions. The mismatched strand includes three base-pair mismatches in each binding domain. As can be seen in Figure 6, the results clearly show a linear response for CoV-DNA in contrast to the mismatched DNA, suggesting that the sandwich hybridization assay is an effective method for selective CoV-DNA detection.

**Figure 6:**
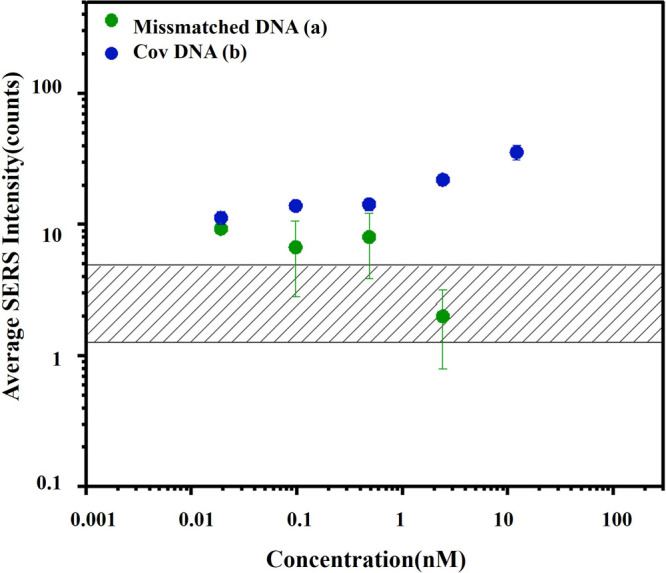
SERS response of the sandwich assay exposed to a) CoV-DNA (in blue dots) with 5 concentrations: 19, 97, 484, 2420, and 12100 nM, and b) mismatch DNA (in green dots) with 4 different concentrations: 19, 97, 484, and 2420 nM. The shaded area represents the signal from the blank sample. Error bars indicate the standard deviation from replicate measurements. All measurements were performed under optimal conditions.

### Assay Development with Binary Digital Raman Map

Quantifying the visual information included in Raman images with image analysis algorithms is becoming the state of the art in the bioanalytical sciences.^37,38^ Here, Raman maps were processed and converted to binary digital Raman maps by using ImageJ software. For digital Raman maps, the threshold was determined based on the mean value of Raman images. The areas below this threshold value are considered background/0 and are displayed in black. Areas above the threshold, corresponding to SERS-active regions, are treated as foreground/1 and are shown as white pixels. Subsequently, using the Analyze menu in ImageJ, we measured the area fraction of the binary digital maps. Area fractions show the percentage of SERS-active area in images. Eventually, the area fraction of each binary Raman map was plotted against different analyte concentrations. As evident in Figure 7 a linear relationship exists between area fraction and various concentrations of the target. With the digital protocol, we are able to define a meaningful region for the SERS nanoprobe intensity by applying thresholds for calculated minimum and observed maximum SERS amounts. This approach aims to reduce the variation in the SERS signal, thereby improving the expected linear fit between the SERS data and the analyte concentration.

**Figure 7:**
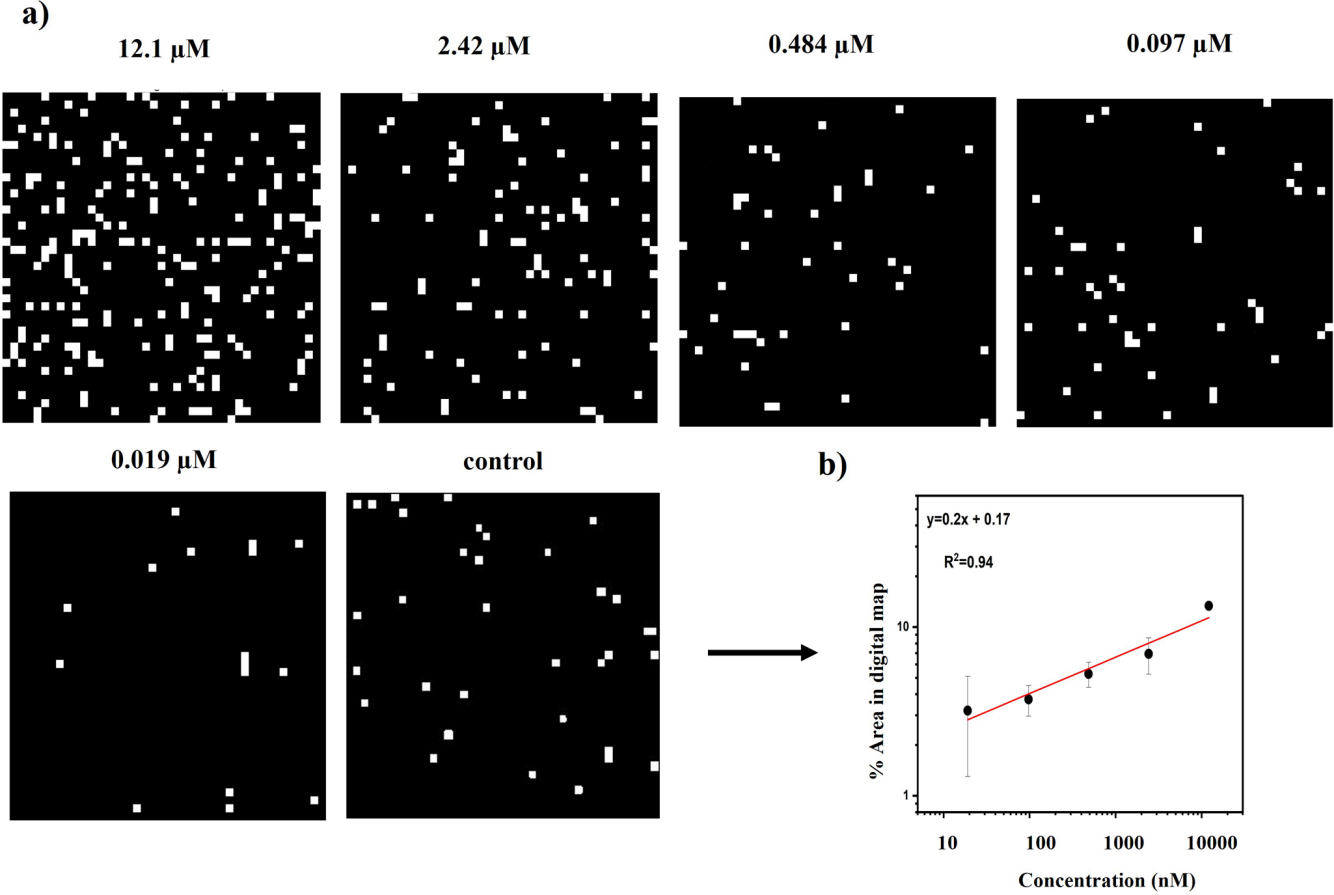
Quantitative detection of CoV-DNA is based on active pixels in binary digital Raman maps. (a) Binary digital Raman maps of DNA concentrations. (b) Logarithmic plot of active pixels in binary digital Raman maps versus different CoV-DNA concentrations.

### Understanding the Mechanism Behind Concentration-Dependent Responses

From the concentration dependency results, we conclude that the SERS signal from the nanoprobes increased with analyte DNA concentration; however, the relationship was not linear. This nonlinearity was also observed in the average number of gold nanoparticles measured using AFM, as well as in both the average intensity and the digital map curves. It is important to note that analytical techniques based on surface-enhanced Raman scattering (SERS), surface plasmon resonance, electrochemistry, or biomolecular interactions often exhibit nonlinear dependencies.^39^ To address this, the concentration dependencies measured by AFM were linearized using semilogarithmic coordinates, while those measured by SERS were linearized using double-logarithmic coordinates.

Therefore, the number of particles anchored to the avidin-coated glass depends on the concentration of analyte DNA as a power function. If we imagine, in a very simplified manner, the process of reporter anchoring as an abstract chemical reaction between the complete sandwich structure and avidin, we can describe the power function exponent (the slope in double-logarithmic coordinates) as a coefficient in front of the analyte DNA in the chemical equation. The equilibrium equation of this reaction links the number of binding nanoparticles (Raman response intensity) with the analyte DNA concentration raised to the power of this exponent. This exponent can be considered an efficiency characteristic of the selective interaction between the analyte DNA and other components of the system, which ultimately leads to successful anchoring on the avidin-coated glass surface.

The increase in the mismatched DNA concentration (Figure 6) did not affect the Raman response efficiency, and all of the data points can be approximated by a horizontal line, which serves as a clear illustration of the above thesis. The formation of the sandwich structure is a complex process that consists of two hybridization events: the binding of CoV-DNA to complementary strands and the deposition of the sandwich structure on the surface via biotin–neutravidin interactions. Each stage can be a source of nonspecific binding, which significantly affects the final result. Based on the obtained results, we propose that nonspecific binding contributes substantially to background formation, thereby limiting the quantification threshold. However, increasing the concentration of noncomplementary macromolecules does not result in the appearance of a concentration curve slope.

It is important to note that the exponent should reflect not only the complementarity of the analyte but also the overall efficiency of the sensing system. In this work, we considered the simplest sandwich-assay technique, where the concentration of analyte DNA alters the binding probability of the Raman reporter to the substrate. Even in this simple system, there are multiple opportunities for improvement and optimization across various areas, including colloidal chemistry: variations in the dye and characteristics of the dye-decorated strand, and changes in the concentrations of components and their ratios (for example, the concentration of nanoparticles and their ratio to dye-labeled strands, or the concentration of biotin-labeled capture strands). Further improvements can be achieved through optimization of the preparation procedure and refinement of the data processing, evaluation, and interpretation. An increase in the slope of the log–log coordinates and a decrease in the background signal of blank samples can be used as markers of method improvement.

## Conclusion

In summary, this study demonstrates the quantitative detection of target DNA using a SERS-based sandwich hybridization assay. The integration of a digital protocol improved the calibration linearity by establishing a threshold for background intensity. This improvement was reflected in a 4.4% increase in the coefficient of determination (R^2^) of the calibration curve, indicating enhanced linearity and quantitative reliability compared with conventional analysis. The assay performance was further validated by correlating the SERS signal with Atomic Force Microscopy (AFM) measurements, yielding a coefficient of determination of 0.99, indicative of strong agreement between the two techniques. Selectivity studies confirmed a concentration-dependent response for the target DNA, while mismatched DNA produced no significant response, with signals overlapping those of the blank. Overall, these findings demonstrate that the SERS-based sandwich hybridization assay, particularly when combined with digital signal analysis, provides a robust, sensitive, and selective platform for bioanalytical applications.

## Supplementary Material



## Notes

Different hybridization conditions (Table S1); histogram including experimental parameter optimization result (Figure S1A–D); evaluation of pixel distribution (Figure S2); AFM digital Raman correlation (Figure S3); and logarithmic plot of SERS intensity against DNA concentration by eq S3 (Figures S4 and S5) (PDF)
