# Supplementary material for: Quantitative SERS-Based Sandwich-Hybridization Assay for Nucleic Acid Detection
Source: ACS Omega. 2025 Oct 28;10(44):52260–8. doi: 10.1021/acsomega.5c01977 (PMC12612968; doi:10.1021/acsomega.5c01977)
Supplement: Supplementary file 1 [file ao5c01977_si_001.pdf]

Supporting Information

Quantitative SERS-Based Sandwich-Hybridization Assay for Nucleic Acid Detection

Kosar Shahsavar[a,b], Amr Mostafa[a], Dina Mahdi-Joest[a], Anton S.Zverev[a], Sergio Kogikoski Jr. [a], Morteza Hosseini[b], Ilko Bald[a*]

[a]*Institute of Chemistry, Hybrid Nanostructures, University of Potsdam, Karl-Liebknecht-Str. 24-25, 14476, Potsdam, Germany.*

[b] *Nanobiosensors Lab, Department of Nanobiotechnology and Biomimetics, School of Life Science Engineering, College of Interdisciplinary Science and Technology, University of Tehran, Tehran 1439817435, Iran*

**Correspondence*: ilko.bald@uni-potsdam.de

Table of Contents

1. **UV–vis Based Concentration Calculation**
2. **Optimization of Biosensor Performance**
3. **Evaluation of Pixel Distribution**
4. **AFM digital Raman correlation**
5. **Understanding the Mechanism Behind Concentration-Dependent Responses**

UV–vis Based Concentration Calculation

The concentration of nanoparticles was determined using UV–vis absorption spectroscopy according to the Beer–Lambert law. Measurements were performed with a NanoDrop™ 2000 spectrophotometer (Thermo Fisher Scientific Inc., Massachusetts, USA). The Beer–Lambert law is given by:

$$A = \varepsilon c l$$

where A is the absorbance, ε is the molar extinction coefficient, c is the molar concentration, and l is the optical path length (0.1 cm). For 40 nm AuNPs, the appropriate ε (8.42×10^{9} M−1·cm−1) was applied, yielding concentrations of 0.14 nM and 0.25 nM before and after DNA coating, respectively.

Optimization of Biosensor Performance

Table S1. *Hybridization condition for sample Ⅰ-Ⅳ*

Sample	Hybridization time (min)	Hybridization Temperature (°C)	Addition of 1x TAE 750 mM NaCl
Ⅰ	10	37	---
Ⅱ	20	37	---
Ⅲ	45	37	---
Ⅳ	20	37	✓

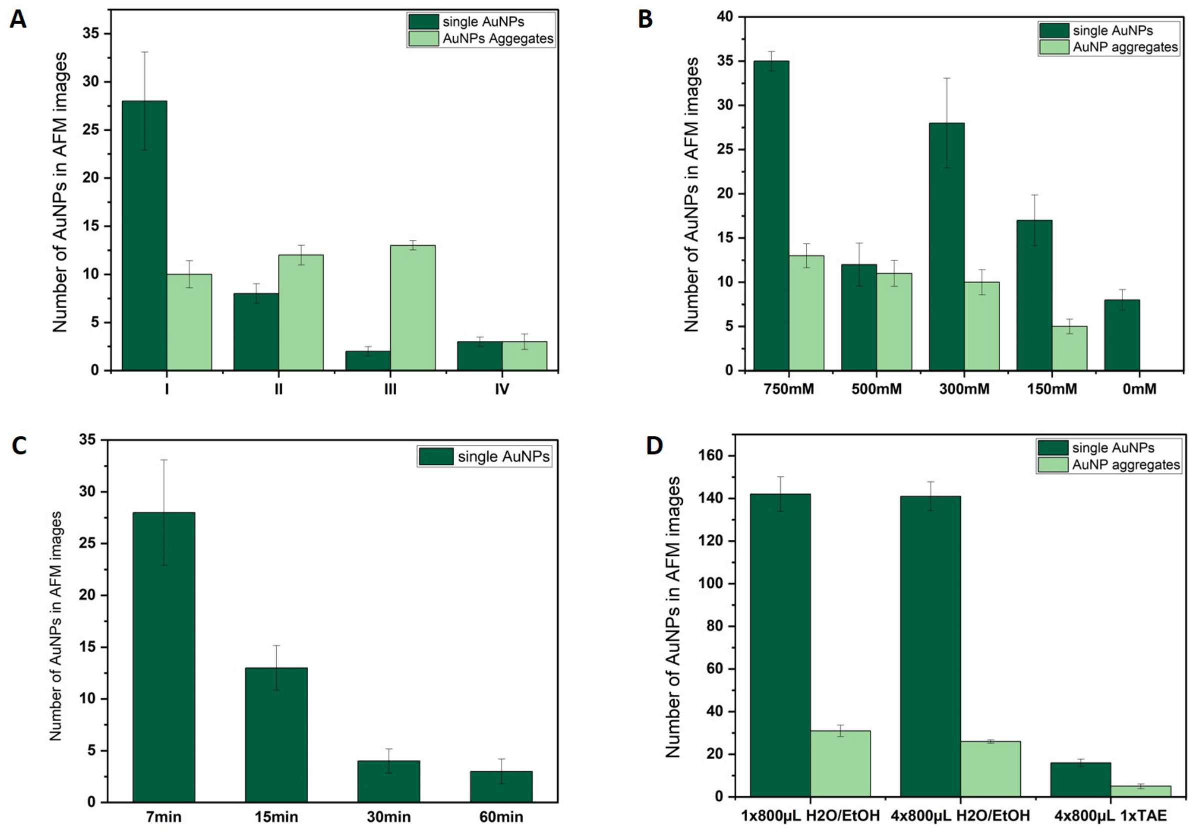

Figure S1. *A) Effect of incubation temperature, time, and ionic strength on the hybridization process. B) Influence of NaCl concentration on the quantity of single AuNPs and AuNP aggregates deposited on the glass. C) Investigation of different incubation times to achieve the highest quantity of single AuNPs. D) Effect of various washing conditions on the amount of single AuNPs and AuNP aggregates.The total counts from five AFM images (each 10×10 µm2) were summed for each condition.Error bars shows the standard deviation (n=5).*

Evaluation of Pixel Distribution

Figure 5 shows the total pixel distribution based on the intensity obtained from SERS mapping at five different concentrations. The shape of the histogram at 0.019 µM shows normal behaviour due to the strong contribution of background signal, but when we are going to higher concentrations, the histogram shape changes to a tailed behaviour. Given that higher concentrations result in a relative decrease in background intensity, this tailed pattern seems reasonable.

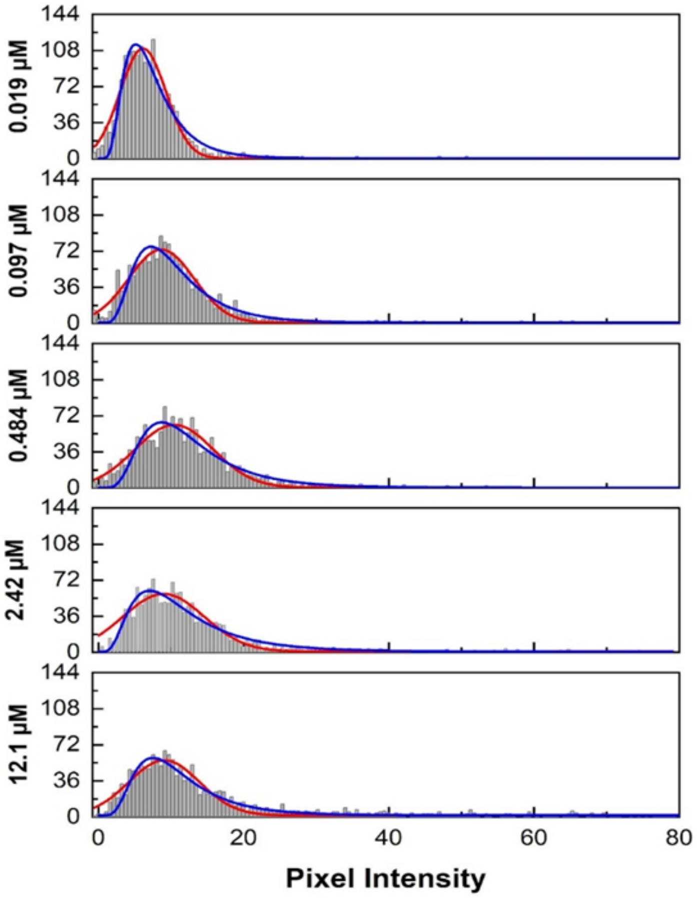

Figure S2. *Distribution plot represents the number of pixels (Y axis) plotted against intensity values (X axis) for the Raman intensity map of each concentration. The blue line is the lognormal fit, and the red curve stands for the Gaussian fit.*

AFM digital Raman Correlation

We tried to correlate the AFM result from each concentration to the SERS outcome of the same concentration in Digital method. The number of particles in square µM^2 (100) from AFM images plotted against the number of pixels per total pixel number (1681) in the digital map. The coefficient of determination (R^2) for the plotted linear regression was 0.98. This result agrees with the theoretically expected outcome that relies on the similarity between the nature of AFM counting and active pixels in a digital map.

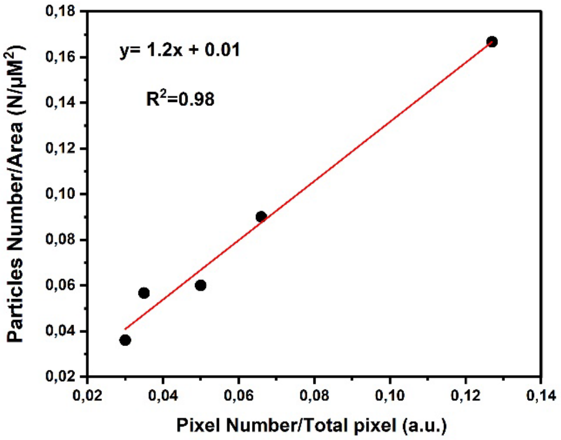

Figure S3. *Linear relationship between the number of single nanoparticles (SERS Nanotag) present on a 100 µM2 surface determined by Atomic Force Microscopy (AFM) and the SERS digital counts obtained from digital Raman map with a size of 200 × 200 µM and a resolution of 5 µM, comprising a total of 1681 pixels.*

Understanding the Mechanism Behind Concentration-Dependent Responses

Assume the suspension of reporter gold nanoparticles (*R*) with concentration C_R, covered by Sandwich-hybridized DNA structures (*S*) and their concentration is equal to the concentration of analyte DNA C_A. Let's suspect that sandwich structures dispersed monotonously through the particles, and each particle has $N=C_A/C_R$ Sandwich structures on the surface. Consider the chemisorption reaction between the sandwich structure and avidin (G). Guess that each sandwich structure reacts individually but binds the whole reporter (GSR) with additional N-1 sandwich structures. Therefore, we can write equation 1, the equilibrium equation for the above reaction. The neutravidin glass surface is big enough to be considered constant. The equilibrium concentration of sandwich structures $[S]=N(C_R-[GSR])$ allows us to write down the final equation 2. We can change the [GSR] on Raman response intensity *I*, and C_R on I_∞ - the proposed Raman response from the whole concentration of reporters to obtain equation 3

$$\text{nS-R} \xrightarrow{\text{Avidin glass}} \text{G-S-R-(S)}_{N-1}$$

$$K = \frac{[GSR]}{[G][S]^n} \qquad \text{eq.1}$$

$$K = \frac{[GSR]}{C_A{}^n(1-\frac{[GSR]}{C_R})^n} \qquad \text{eq.2}$$

$$K = \frac{I}{C_A{}^n (1 - \frac{I}{I_\infty})^n} \qquad \text{eq.3}$$

The approximation of experimental concentration curve by eq. 3 is shown in fig S5. Approximation curves and experimental dots are also approximated by power functions, which have quite similar parameters. This fact shows that using by usual power function is in general enough for approximation of the experiment.

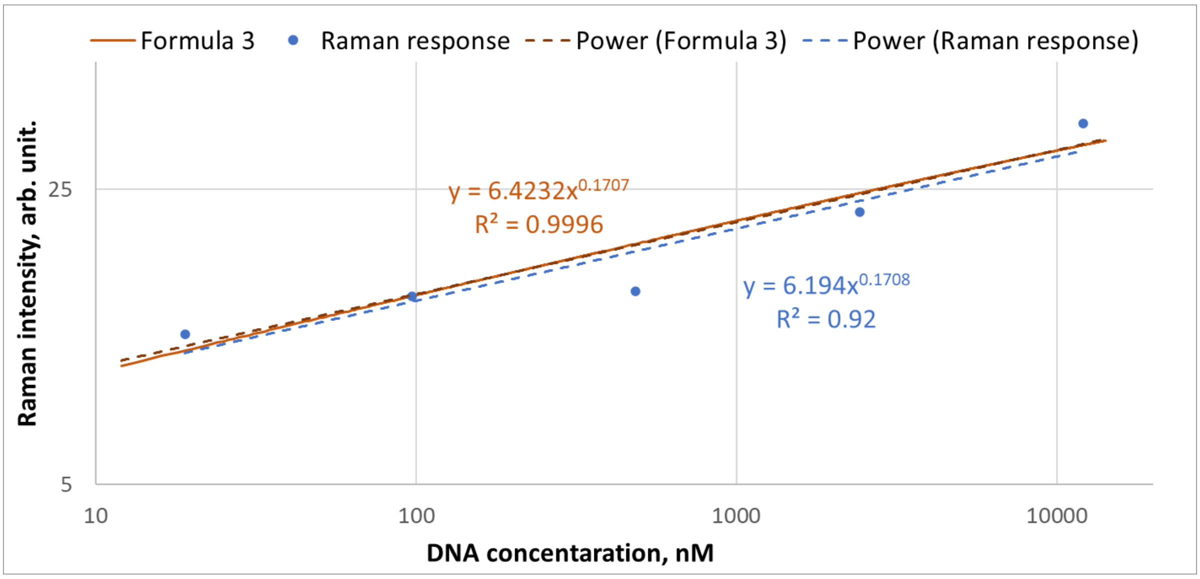

Figure S4. *Logarithmic plot of SERS intensity of the characteristic peak at 1652 cm-1 against CoV-DNA concentration approximated by eq. 3 with K=6.15, n=0.19, and I_∞=60*

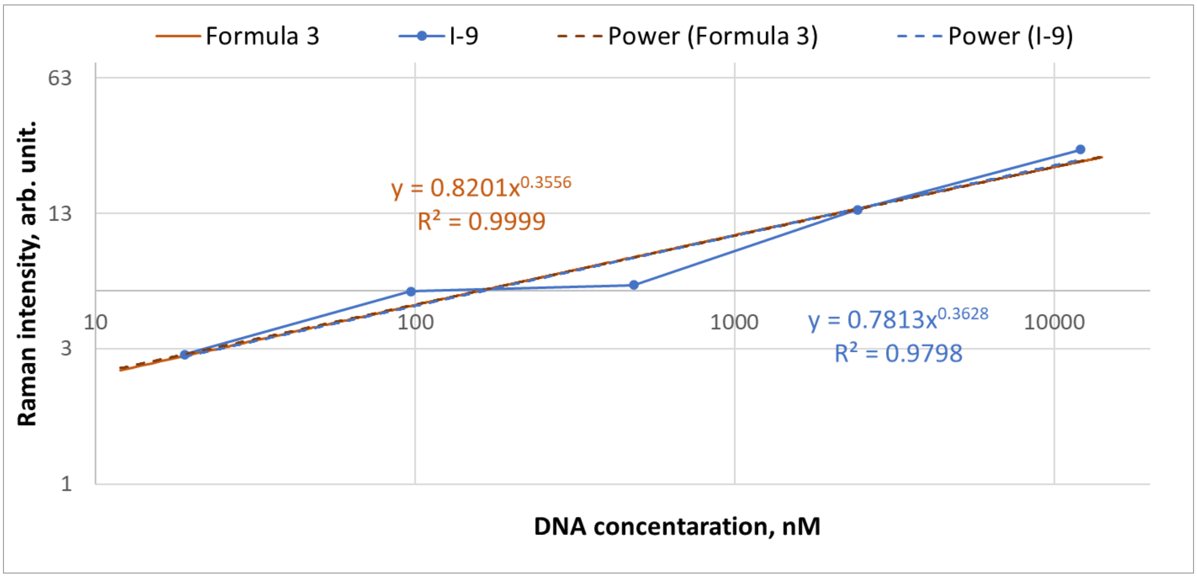

Figure S5. *Logarithmic plot of SERS intensity of the characteristic peak at 1652 cm-1 against CoV-DNA concentration approximated by eq. 3 with K=0.78, n=0.368, and I_∞=130*
